# The Oxysterol 24*(S)*,25-Epoxycholesterol Attenuates Human Smooth Muscle–Derived Foam Cell Formation Via Reduced Low-Density Lipoprotein Uptake and Enhanced Cholesterol Efflux

**DOI:** 10.1161/JAHA.112.000810

**Published:** 2012-06-22

**Authors:** Michael M. Beyea, Samantha Reaume, Cynthia G. Sawyez, Jane Y. Edwards, Caroline O'Neil, Robert A Hegele, J Geoffrey Pickering, Murray W Huff

**Affiliations:** Robarts Research Institute, Schulich School of Medicine and Dentistry, University of Western Ontario, London, Canada (M.M.B., C.G.S., J.Y.E., C.O., R.A.H., J.G.P., M.W.H); Department of Medicine, Schulich School of Medicine and Dentistry, University of Western Ontario, London, Canada (C.G.S., J.Y.E., R.A.H., J.G.P., M.W.H); Department of Biochemistry, Schulich School of Medicine and Dentistry, University of Western Ontario, London, Canada (M.M.B., S.R., R.A.H., J.G.P., M.W.H.)

**Keywords:** vascular smooth muscle cell, lipoproteins, oxysterol, liver X receptor, cholesterol efflux

## Abstract

**Background:**

Foam cell formation by intimal smooth muscle cells (SMCs) inhibits the elaboration of extracellular matrix, which is detrimental to plaque stabilization. In the present study, we examined the lipoproteins and receptors involved in human SMC foam cell formation and investigated the ability of 24(*S*),25-epoxycholesterol [24(*S*),25-EC], an oxysterol agonist of the liver X receptor, to attenuate SMC foam cell formation.

**Methods and Results:**

Incubation of human internal thoracic SMCs with atherogenic lipoproteins demonstrated that low-density lipoprotein (LDL), but not oxidized or acetylated LDL, was the primary lipoprotein taken up, resulting in marked cholesteryl ester deposition (6-fold vs 1.8-fold; *P*<0.05; n=4). Exposure of SMCs to exogenous or endogenously synthesized 24*(S)*,25-EC attenuated LDL uptake (−90% and −47% respectively; *P*<0.05; n=3) through decreased *sterol regulatory element–binding protein-2* expression (−30% and −17%, respectively; *P*<0.001; n=3), decreased *LDL receptor* expression (−75% and −40%, respectively; *P*<0.05; n=3) and increased liver X receptor–mediated *myosin regulatory light chain interacting protein* expression (7- and 3-fold, respectively; *P*<0.05; n=4). Furthermore, exogenous 24*(S)*,25-EC increased adenosine triphosphate–binding cassettes A1– and G1–mediated cholesterol efflux to apolipoprotein AI (1.9-fold; *P*<0.001; n=5) and high-density lipoprotein_3_ (1.3-fold; *P*<0.05; n=5). 24*(S)*,25-EC, unlike a nonsteroidal liver X receptor agonist, T0901317, did not stimulate *sterol regulatory element–binding protein-1c*–mediated fatty acid synthesis or triglyceride accumulation. 24(*S*),25-EC preserved the assembly of fibronectin and type I collagen by SMCs.

**Conclusions:**

The oxysterol 24*(S)*,25-EC prevented foam cell formation in human SMCs by attenuation of LDL receptor–mediated LDL uptake and stimulation of cholesterol efflux, restoring the elaboration of extracellular matrix. In contrast to T0901317, 24*(S)*,25-EC prevented the development of a triglyceride-rich foam cell phenotype. **(*J Am Heart Assoc*. 2012;1:e000810 doi: 10.1161/JAHA.112.000810.)**

## Introduction

Human smooth muscle cells (SMCs) play an integral role in atherogenesis.^1^ SMCs are stimulated to migrate and proliferate within the intima in response to cytokines and growth factors.^1,2^ SMCs in culture can accumulate lipoprotein-derived lipids and adopt a foam cell–like phenotype.^3,4^ Furthermore, atherosclerotic lesions contain abundant foam cells of SMC origin.^1^ A vital role of vascular SMCs is to stabilize lesions through the elaboration of collagen fibrils, which protects from plaque rupture.^1^ However, SMCs that assume the foam cell state are potentially detrimental to plaque stability. Recently, we reported that lipid accumulation renders cultured human SMCs incapable of efficiently assembling a fibrillar extracellular matrix.^5^ Therefore, elucidation of potential mechanisms for the prevention of SMC foam cell formation has implications for plaque stability and ultimately for clinical events.

There is still uncertainty as to which atherogenic lipoproteins stimulate human SMC foam cell formation and what mechanisms are involved in their uptake. Furthermore, SMCs display phenotypic diversity,^6^ and evidence has emerged that human SMC subtypes demonstrate distinct responses to atherogenic lipoproteins.^5^ In contrast to macrophages, rabbit aortic SMC foam cell formation was induced primarily by uptake of native low-density lipoprotein (LDL) but not modified LDL.^3,4^ We previously demonstrated that human SMCs isolated from the internal thoracic artery readily accumulated lipids when exposed to human LDL and very-low-density lipoprotein (VLDL) but resisted lipid accumulation when exposed to modified lipoproteins.^7^ Whether this difference in lipoprotein uptake was related to differential expression of lipoprotein receptors has not been established. Furthermore, the larger, slower-growing, spindle-shaped SMC clone accumulated more lipid in response to lipoprotein exposure than did the smaller, faster-growing, epithelioid-shaped SMC clone,^7^ which suggests that the former cell type represents the predominant SMC foam cell precursor.

The liver X receptor (LXR) is an important regulator of cholesterol homeostasis.^8^ In macrophages, LXR activation by endogenous agonists, including oxysterols, or by exogenous synthetic nonsteroidal compounds, such as T0901317, activated the transcription of genes involved in cholesterol efflux, including *adenosine triphosphate–binding cassette (ABC) A1* and *ABCG1*.^8^ T0901317 decreased aortic lesions in mouse models of atherosclerosis, mediated in part by attenuation of macrophage foam cell formation through enhanced cholesterol efflux.^9,10^ Given the abundance of SMC-derived foam cells in atherosclerotic lesions,^1^ LXR agonist–stimulated cholesterol efflux in SMCs could also contribute to atheroprotection. Characterization of LXR activation in SMCs remains incomplete. Although T0901317 stimulated ABCA1- and ABCG1-mediated cholesterol efflux in human SMCs,^11,12^ the impact of LXR activation on atherogenic lipoprotein uptake and lipid deposition has not been explored. Triglyceride has also been shown to contribute to foam cell formation, particularly in the setting of elevated plasma VLDL and free fatty acids (FA), a profile typical of the metabolic syndrome and diabetes.^13^ An adverse effect of nonsteroidal LXR agonists, including T0901317, is increased triglyceride accumulation, induced by transactivation of genes involved in FA synthesis, including *sterol regulatory element–binding protein* (*SREBP*)*-1c* and *fatty acid synthase (FASN)*.^14^ In human SMCs, although T0901317 induced cholesterol efflux,^11^ it stimulated *SREBP-1c*–induced lipogenesis and triglyceride accumulation in cells exposed to adipogeneic differentiation medium.^13^

In macrophages, natural oxysterol LXR ligands such as 24(*S*),25-epoxycholesterol [24(*S*),25-EC], added either exogenously or synthesized endogenously, selectively activated ABCA1- and ABCG1-mediated cholesterol efflux.^15,16^ In contrast to T0901317, 24*(S),*25-EC or another oxysterol-like LXR agonist (DMHCA; N,N-dimethyl-3β-hydroxy-cholenamide) had little effect on *SREBP-1c–* or *FASN*-stimulated lipogenesis or triglyceride accumulation.^15–17^ Selectivity of 24*(S)*,25-EC was attributed to inhibition of precursor (p)SREBP-1c processing to its active nuclear form (nSREBP-1c) by the oxysterol but not by T0901317, thus preventing lipogenic gene expression.^15,16^

Oxysterols also disrupt the maturation of SREBP-2. SREBP-2 stimulated the expression of genes involved in de novo cholesterol synthesis and lipoprotein uptake, including *3-hydroxy-3-methylglutaryl-coenzyme A reductase (HMGCR)* and the *LDL receptor (LDLR),* respectively.^18^ In Chinese hamster ovary cells, 24*(S)*,25-EC (like other oxysterols) blocked the maturation of pSREBP-2, similar to the effect observed for SREBP-1c.^18,19^ Whether 24*(S)*,25-EC attenuates cholesterol accumulation in SMCs through SREBP-2–regulated gene expression has not been reported.

The objectives of the present study were 2-fold: (1) to identify the receptors responsible for lipoprotein uptake in human vascular SMCs, and (2) to examine the impact of 24*(S)*,25-EC on SMC foam cell formation and the elaboration of extracellular matrix. We studied a clonal population of SMCs—namely, human internal thoracic (HIT) C6, which adopts a foam cell phenotype after exposure to atherogenic lipoproteins.^7^ We hypothesized that exogenous 24*(S)*,25-EC or endogenously synthesized 24*(S)*,25-EC attenuates lipoprotein-induced cholesteryl ester (CE) accumulation in SMCs, without altering triglyceride synthesis.

We demonstrate that LDL, but not modified LDL, preferentially induced SMC foam cell formation. Exogenous or endogenously synthesized 24*(S)*,25-EC reduced SMC uptake of native LDL by inhibiting SREBP-2–mediated *LDLR* expression. Concurrently, 24*(S)*,25-EC activated ABCA1- and ABCG1-mediated cholesterol efflux but did not stimulate SREBP-1c–induced lipogenesis or triglyceride accumulation. These experiments reveal a dual role for 24*(S)*,25-EC in attenuating CE accumulation in human SMCs: reduced LDL uptake and enhanced cholesterol efflux, resulting in preservation of SMC assembly of extracellular matrix.

## Methods

### Cell Culture

The HIT-SMC clone used in this study, C6, is a human clonal line derived from the media of distal internal thoracic artery, as described previously.^6,20^ SMCs were cultured in M199 media with 10% fetal bovine serum (FBS; Invitrogen, Burlington, ON) until 100% confluent and were seeded into 100-mm or 35-mm tissue culture dishes (BD Falcon, Franklin Lakes, NJ) at 7000 cells/cm^2^ for experiments.^7^ Unless otherwise indicated, cells were then preincubated for 24 h in M199 media containing 0.4% FBS (basal), followed by a 24-h incubation with the indicated concentrations of 24*(S)*,25-EC (Steraloids, Inc, Newport, RI) dissolved in 100% ethanol, T0901317 (Sigma, Oakville, ON) dissolved in dimethyl sulfoxide (Sigma), or an inhibitor of 2,3-oxidosqualene:lanosterolcyclase (OSCi) (RO-0714565; F. Hoffmann–La Roche AG Pharmaceuticals Division, Basal, Switzerland) dissolved in dimethyl sulfoxide. No significant effect of 24*(S)*,25-EC, OSCi, or T0901317 on total cellular protein was observed at the concentrations tested (data not shown).

24*(S)*,25-EC is a potent LXR ligand and is uniquely derived from a shunt in the cholesterol biosynthetic pathway (reviewed in Huff et al ^21^). 2,3-Oxidosqualene:lanosterol cyclase (OSC), a cholesterol biosynthesis enzyme downstream of 3-hydroxy-3-methylglutaryl–coenzyme A reductase, catalyzes the conversion of 2,3-monoepoxysqualene to lanosterol, the dedicated step in cholesterol biosynthesis. If 2,3-monoepoxysqualene builds up, it is converted to 2,3;22,23-diepoxysqualene by squalene epoxidase. However, OSC also catalyzes cyclization of 2,3;22,23-diepoxysqu- alene to 24*(S)*,25-epoxylanosterol, which is subsequently transformed into 24*(S)*,25-EC. Synthesis of 24*(S)*, 25-EC is favored over cholesterol under conditions of partial OSC inhibition because of the higher affinity of OSC for 2,3;22,23-diepoxysqualene than for 2,3-monoepoxysqualene. Greater inhibition of OSC eventually results in the inhibition of both 24*(S)*,25-EC and cholesterol.^21^

### Lipoprotein Isolation

VLDL, LDL, and high-density lipoprotein_3_ (HDL_3_) were isolatedfrom plasma of human subjects recruited from the Lipid Clinic at the London Health Sciences Centre, University Campus (London, Ontario, Canada). This study was approved by the University of Western Ontario Institutional Review Board (protocol No. 15685). Lipoproteins were separated by differential ultracentrifugation as described previously.^22^ LDL was oxidized (oxLDL) via the copper sulfate method^22^ or was acetylated (acLDL) by using acetic anhydride,^23^ and the extent of modification was confirmed by alterations in electrophoretic mobility.^22^

### Microscopy of Lipid-Loaded SMCs

Fluorescence micrographs of HITC6 human vascular SMCs were obtained in cells preincubated for 24 h in M199 media containing 0.4% FBS, followed by a further 24-h incubation in M199 media plus 0.4% FBS with or without LDL (150 μg lipoprotein cholesterol/1 mL media) or VLDL (50 μg lipoprotein cholesterol/1 mL media). Paraformaldehyde-fixed cells were mounted in PermaFluor (Thermo Electron Corporation) containing Hoechst 33258 (2.5 μg/mL, Sigma), were stained with boron-dipyrromethene 493/503 (1 μg/mL, Molecular Probes, Eugene, OR), and were visualized by fluorescence microscopy at 493-nm excitation to identify droplets of neutral lipid.^5^

### Assessment of SMC-Mediated Fibronectin and Collagen Assembly

HITC6 human vascular SMCs, plated in 35-mm tissue culture dishes, were preincubated for 24 h in 0.4% FBS media and then incubated for another 24 h with the addition of either vehicle or 24*(S)*,25-EC (1 μmol/L). For a further 24 h, cells were incubated in fresh 0.4% FBS media plus vehicle or 24*(S)*,25-EC with or without LDL (150 μg lipoprotein cholesterol/1 mL media) and Oregon Green–labeled human fibronectin (100 nmol/L) or Oregon Green–labeled rat tail collagen (1 μg/mL). Paraformaldehyde-fixed cells were stained with Oil Red O to identify neutral lipid droplets, and nuclei were counterstained with Hoechst 33258 (2.5 μg/mL; Sigma).

### Cellular Lipid Mass

Lipid mass was determined in SMCs preincubated in media plus 0.4% FBS for 24 h (6-well [35-mm] plates), followed by a further 24-h incubation in the same media with vehicle, LXR agonists, or OSCi (compounds). Cells were then incubated in fresh M199 media plus 0.4% FBS with or without compounds and with or without 150 μg/mL (micrograms lipoprotein cholesterol per milliliter media) of native LDL, acLDL, or oxLDL or 50 μg/mL (micrograms lipoprotein cholesterol per milliliter media) VLDL plus 0.1 U/mL of bovine lipoprotein lipase.^7^ Lipids were extracted with hexane:isopropanol (3:2 volume:volume) and processed for lipid quantification as described previously.^22^ Cell protein was assayed after solubilization in 0.1 N sodium hydroxide. Total cholesterol (TC), free cholesterol (Wako Chemicals, Richmond, VA), and triglyceride (Roche Diagnostics, Inc, Indianapolis, IN) were determined enzymatically by using commercial reagents as described previously.^22^ CE mass was calculated by subtracting free cholesterol from TC. Values were normalized to total cell protein.

### Cholesterol Esterification and Cholesterol, 24*(S)*,25-EC, Fatty Acid, and Triglyceride Synthesis

Confluent (80%) SMCs were preincubated in 6-well plates for 24 h in M199 media plus 0.4% FBS and then in the same media with or without compounds for a further 24 h. Subsequently, lipoproteins (LDL and acLDL, 150 μg/mL [micrograms lipoprotein cholesterol per milliliter media]; VLDL, 50 μg/mL [micrograms lipoprotein cholesterol per milliliter media]) plus radiolabeled [1-^14^C]-oleic acid complexed with FA-free bovine serum albumin (FAF:BSA) were added for 5 h in fresh M199 media plus 0.4% FBS, with or without compounds. Lipids were extracted (as described above) and separated via thin-layer chromatography, and radiolabel incorporation into CE and triglyceride was determined according to previously described methods.^16,24^ The synthesis of cholesterol and FA was determined in SMCs preincubated 24 h in media plus 0.4% FBS and then with vehicle, 24*(S)*,25-EC, OSCi, or T0901317 for a further 24 h.[1-^14^C]-acetate with or without compounds in fresh M199 media plus 0.4% FBS was added for an additional 5 h.^16^ The synthesis of 24*(S)*,25-EC was determined in SMCs preincubated for 24 h in M199 0.4% FBS media followed by a 24-h incubation with the OSCi (0 to 100 nmol/L) and [1-^14^C]-acetate in fresh M199 media plus 0.4% FBS. Lipid extraction, saponification, thin-layer chromatography separation, and the amount of radiolabel incorporation into cholesterol, 24*(S)*,25-EC, and FA were determined as described previously.^16^ Values were normalized to total cell protein.

### Lipoprotein Uptake

LDL and acLDL uptake was determined by using fluorescently labeled native and modified 1,1’-dioctadecyl-3,3,3’,3’-tetramethylindocarbocyanine perchlorate (DiI)–labeled lipoproteins (Biomedical Technologies, Inc, Stoughton, MA). Confluent (80%) SMCs in 6-well (35-mm) plates were preincubated in M199 media plus 0.4% FBS for 24 h and then were incubated with or without the addition of vehicle or compounds for a further 24 h. Subsequently, cells were incubated in fresh media and compounds in the presence or absence of 10 μg/mL DiI-LDL or DiI-acLDL for 5 h. Cells were trypsinized and resuspended in phosphate-buffered saline for analysis by flow cytometry. SMCs were excited in the flow chamber of a 2-laser BD FACSCalibur (BD Biosciences, Franklin Lakes, NJ) using the 15-mW, 488-nm, air-cooled, argon-ion laser, and emission spectra were detected in the FL2 (PE/PI) channel (585/42 band-pass filter). Data were collected and analyzed with BD Cell Quest Pro Software.

### Cholesterol Efflux

Cholesterol efflux was determined according to methods described previously.^15,16^ SMCs (80% confluent) were initially incubated in M199 media containing 0.4% FBS, followed by incubation with fresh M199 media containing FAF:BSA (0.2%) plus the addition of acLDL (5 μg TC) and 1 μCi of [^3^H]-cholesterol (Amersham Biosciences, Oakville, ON) per 1 mL of 0.2% FAF:BSA media to label endogenous cholesterol pools, for 24 h. Cells were then washed and incubated with fresh M199 0.2% FAF:BSA media with or without compounds for 24 h, followed by incubation with fresh M199 0.2% FAF:BSA media with or withoutcompounds and with or without apolipoprotein (apo) A1 (10 μg/mL) or HDL_3_ (100 μg/mL) for 16 h. Radioactivity was measured in aliquots of media and cell lysate by using a scintillation counter and was expressed as a percent of total radioactivity per well (media plus cell lysate). The percentage of cholesterol effluxed was normalized to total cell protein.

### Real-Time Quantitative Reverse Transcription Polymerase Chain Reaction

Total RNA from confluent (80%) SMCs was isolated by using Trizol reagent (Invitrogen, Burlington, ON) after 24-h preincubation in 100-mm dishes in M199 media plus 0.4% FBS, followed by a further 24 h in the same media with or without compounds, as described above. RNA (2 μg) was reverse-transcribed as described previously,^16^ and mRNA abundances were determined by a 2-step quantitative real-time reverse transcription polymerase chain reaction method on an ABI Prism (model 7900HT) Sequence Detection System (Applied Biosystems, Foster City, CA), according to the manufacturer's instructions. cDNA (20 ng) was analyzed in triplicate for *ABCA1, ABCG1, CD36, FASN, HMGCR, LDLR, LXRα, proprotein convertase subtilisin/kexin type 9* (*PCSK9*), *myosin regulatory light chain interacting protein (MYLIP;* also known as *IDOL [inducible degrader of the LDL receptor], scavenger receptor AI/II (SRAI/II), scavenger receptor class B1 (SR-B1), SREBP-1c,* and *SREBP-2*. For each gene, the standard curve method was used to determine mRNA abundance, which was normalized to the abundance of *glyceraldehyde 3-phosphate dehydrogenase* *(GAPDH)*. Quantities of mRNA for a specific gene were interpolated from a standard curve plotted as cycle threshold versus the log of the quantity of serial dilutions of a known cDNA standard. This method also allowed for the comparison of basal expression of lipoprotein receptors relative to the *LDLR*. Primer and probe sets for each gene were obtained from Taqman Assays-on-Demand (Applied Biosystems), with the exception of *SREBP-1c,* which was designed as described previously to differentiate the 2 novel exon 1 variants generated from alternative start sites.^16^

### Immunoblotting

SMCs (80% confluent) were plated in 6-well (35-mm) plates in triplicate, were preincubated in M199 media plus 0.4% FBS, and subsequently were incubated for a further 24 h in the same media with or without compounds. SMC lysates (10 mmol/L Tris-HCl, 10 mmol/L NaCl, 3 mmol/L MgCl_2_, 0.5% Igepal, pH 7.4, containing protease inhibitor and phosphatase inhibitor cocktail according to the manufacturer's instructions [Sigma-Aldrich, Oakville, ON]) were homogenized by passes through a 25-gauge needle. The postnuclear supernatant was obtained by centrifugation (500*g*, 4°C, 10 minutes). The remaining pellet was washed once with lysis buffer, was resuspended in hypertonic buffer (10 mmol/L Hepes, 0.42 mol/L NaCl, 1.5 mmol/L MgCl_2_, 1 mmol/L EDTA, 1 mmol/L EGTA, 1 mmol/L DTT) containing protease and phosphatase inhibitors (as above), and was incubated at 4°C for 30 minutes. The nuclear fraction was obtained by centrifugation (100 000*g*, 4°C, 30 minutes). Protein from the postnuclear or nuclear fractions was mixed with sodium dodecyl sulfate loading buffer and subjected to sodium dodecyl sulfate polyacrylamide gel electrophoresis on either an 8% (ABCA1, ABCG1, and LDLR, 20 μg or 10 μg postnuclear fraction) or 10% (SREBP-1, 30 μg nuclear and 20 μg postnuclear fractions) gel. Proteins were transferred to polyvinylidene fluoride membranes and probed with rabbit polyclonal anti-ABCA1, rabbit polyclonal anti-ABCG1 (Novus Biologicals, Littleton, CO), chicken polyclonal anti–human LDL receptor (Abcam, Inc, Cambridge, MA), or mouse monoclonal anti–SREBP-1 (Neomarkers, Fremont, CA), followed by the appropriate secondary antibody conjugated to horseradish peroxidase (Santa Cruz Biotechnology, Inc, Santa Cruz, CA) for development with chemiluminescence substrate. Membranes were stripped and reprobed with a polyclonal anti–β-actin antibody (Cell Signaling, Danvers, MA) for postnuclear fractions and Lamin A/C (Santa Cruz Biotechnology, Inc, Santa Cruz, CA) for nuclear fractions for use as loading controls. Band intensities were quantified with an imaging densitometer as described previously.^16^

### Statistical Analysis

Each experimental method was performed with biological replicates of 3 to 5 (n=3–5), with 3 to 4 technical replicates within each biological replicate. Graphical results are presented as the mean ± standard error of the mean (SEM). The Shapiro-Wilk normality test was used to test for parametric distributions in each data set. *P* values for observed differences between treatment and control groups were calculatedwith the Student *t* test for parametric data or the Mann-Whitney *U* test for nonparametric data. Statistically significant observations were defined by a 2-tailed threshold of *P*<0.05. Statistical analyses were performed with SigmaPlot 11.0 software (Systat, Inc, San Jose, CA).

## Results

### 24*(S)*,25-EC, OSCi, and T0901317 Attenuate LDL Uptake, CE Mass, and Cholesterol Esterification in SMCs

**Figure 1.**
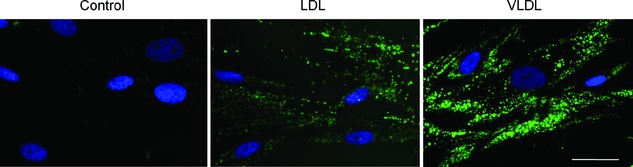
Generation of lipid-laden human vascular SMCs incubated with human LDL or VLDL. Fluorescence micrographs of HITC6 human vascular SMCs incubated for 24 h in M199 media containing 0.4% FBS followed by a 24-h incubation with human LDL (150 μg cholesterol / mL media) or human VLDL (50 μg cholesterol / mL media). Paraformaldehyde-fixed cells were mounted in PermaFluor (Thermo Electron Corporation) containing Hoechst 33258 and were stained with boron-dipyrromethene 493/503 to identify droplets of neutral lipid. The images illustrate abundant cytoplasmic lipid droplets in LDL- and VLDL-exposed SMCs. Bar=25 μm. Images are representative of 3 separate experiments.

**Figure 2.**
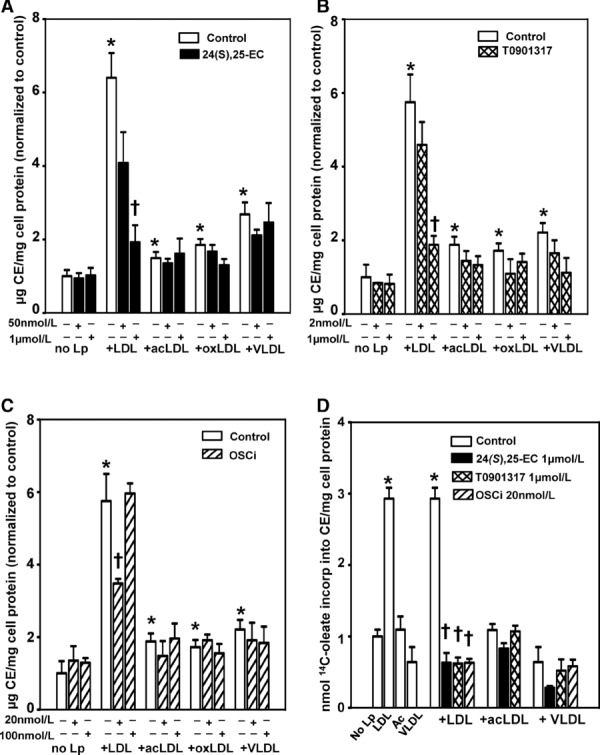
24*(S)*,25-EC, T0901317, and OSCi attenuate LDL-induced CE mass and cholesterol esterification in human SMCs. CE (A, B, and C) and cholesterol esterification (D) in SMCs incubated in M199 media containing 0.4% FBS (24 h); followed by an incubation in M199 0.4% FBS with or without vehicle, 24*(S)*,25-EC, T0901317, or OSCi for 24 h; followed by incubation in M199 0.4% FBS with or without compounds and with or without LDL, acLDL, or oxLDL (150 μg lipoprotein cholesterol / mL media) or VLDL (50 μg lipoprotein cholesterol / mL media), or [^14^C]oleate (D) for 16 h (A through C) or 5 h (D). Values are mean±SEM for 4 separate experiments (n=4). *P<0.05 vs no lipoprotein (No Lp) and †*P*<0.05 vs control plus LDL, as analyzed by the nonparametric Mann-Whitney *U* test.

**Figure 3.**
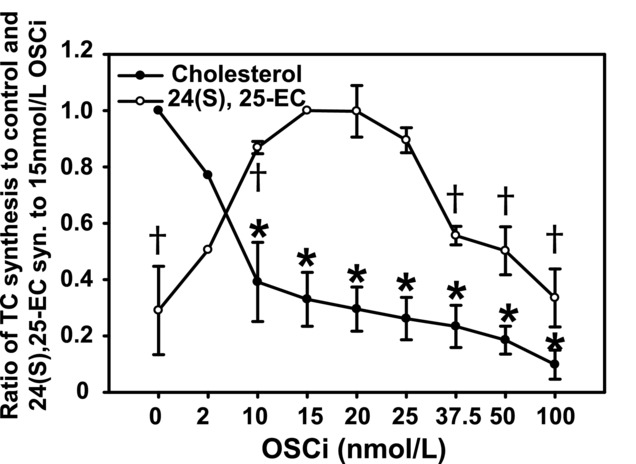
Cholesterol and 24*(S)*,25-EC synthesis in human SMCs incubated with increasing concentrations of OSCi. HITC6 SMCs were preincubated in M199 media containing 0.4% FBS (24 h) and then were incubated with vehicle or OSCi plus [^14^C]-acetate for 24 h. Acetate incorporation into cholesterol and 24*(S)*,25-EC is calculated as disintegrations per minute per milligram of cell protein and is expressed for cholesterol as percent of vehicle alone (mean±SEM) and for 24(*S*),25-EC as percent of the 15 nmol/L OSCi (mean±SEM), for 3 separate experiments (n=3). *P<0.05 vs vehicle alone for cholesterol synthesis and †*P*<0.05 vs 15 nmol/L OSCi for 24*(S),*25-EC, analyzed by the Student *t* test.

SMC foam cells were generated by exposure to human lipoproteins and yielded cells engorged with lipid droplets (Figure 1). LDL significantly increased CE mass 6-fold and cholesterol esterification 3-fold (*P*<0.05) (Figure 2A through 2D). Modified LDL, both acLDL and oxLDL, and native VLDL increased CE mass 1.5- to 2.5-fold (*P*<0.05 for each) (Figure 2A through 2C) but did not affect cholesterol esterification (*P*=NS) (Figure 2D). Preincubation with either 24*(S)*,25-EC or T0901317 at 1 μmol/L significantly reduced LDL-mediated CE accumulation by ≈70% (*P*<0.05 for both) (Figure 2A and 2B) and prevented the LDL-induced increase in cholesterol esterification (Figure 2D). Endogenous synthesis of 24*(S)*,25-EC, induced by the OSCi, decreased LDL-stimulated CE in a biphasic manner. The OSCi at 20 nmol/L decreased CE mass by 35% (*P*<0.05) (Figure 2C), which coincided with maximal 24*(S)*,25-EC synthesis (Figure 3). The OSCi at 100 nmol/L, which did not stimulate 24*(S)*,25-EC synthesis, did not decrease CE mass (*P*=NS for both). At 20 nmol/L, the OSCi prevented LDL-induced cholesterol esterification (*P*=NS) (Figure 2D). The modest increases in CE mass induced by acLDL, oxLDL, or VLDL (1.5- to 2-fold, *P*<0.05 for each) were unaffected by 24*(S)*,25-EC, T0901317, or the OSCi (20 nmol/L) (*P*=NS, compared to lipid-loaded control) (Figure 2A through 2C), and cholesterol esterification rates in SMCs incubated with acLDL or VLDL were unchanged (*P*=NS for both). Free cholesterol mass was unaltered by any treatment (data not shown).

### 24*(S)*,25-EC and OSCi Decrease LDLR Expression but Do Not Affect Scavenger Receptor Expression in SMCs

**Figure 4.**
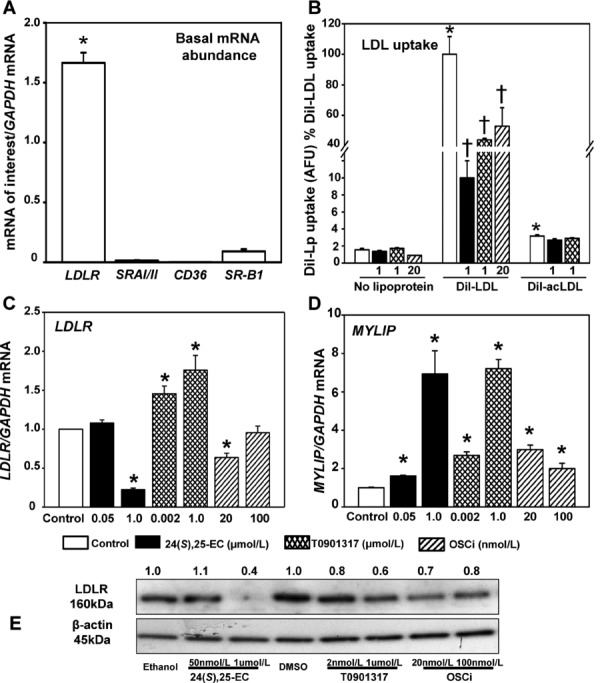
24*(S)*,25-EC and OSCi reduce LDL uptake into human SMCs through decreased expression of *LDLR*. A, mRNA of *LDLR, SRAI/II, CD36,* and *SR-B1* normalized to *GAPDH* from SMCs, incubated in M199 media containing 0.4% FBS (basal conditions) (48 h). Values are mean±SEM for 3 separate experiments (n=3). *P<0.03 vs *SRAI/II, CD36*, and *SR-B1,* analyzed by the Mann-Whitney *U* nonparametric test. B, SMCs were incubated in M199 0.4% FBS (24 h) and then with the same media with or without vehicle, 24*(S)*,25-EC, T0901317, or OSCi (24 h), followed by a 5-h incubation in M199 0.4% FBS with or without vehicle alone, DiI-LDL, or DiI-acLDL (10 μg/mL). Uptake was determined by flow cytometry. Values are mean±SEM for 3 separate experiments (n=3). *P<0.01 vs no lipoprotein and †*P*<0.02 vs DiI-LDL, analyzed by Student *t* test. C and D, mRNA of *LDLR* (C) and *MYLIP* (D)*,* normalized to *GAPDH* from SMCs incubated in M199 0.4% FBS media (24 h), followed by a 24-h incubation in M199 0.4% FBS media with or without vehicle, 24*(S)*,25-EC, T0901317, or OSCi. Key showing bar fill patterns applies to panels B, C, and D. Values are mean±SEM, relative to control, for 4 separate experiments (n=4). *P<0.03 vs vehicle analyzed by the Mann-Whitney *U* nonparametric test. E, Representative immunoblot of LDL receptor from postnuclear fractions of SMC incubated with compounds as in C and D. Quantification is the mean of band intensities normalized to β-actin from 4 separate experiments. DMSO indicates dimethyl sulfoxide.

**Figure 5.**
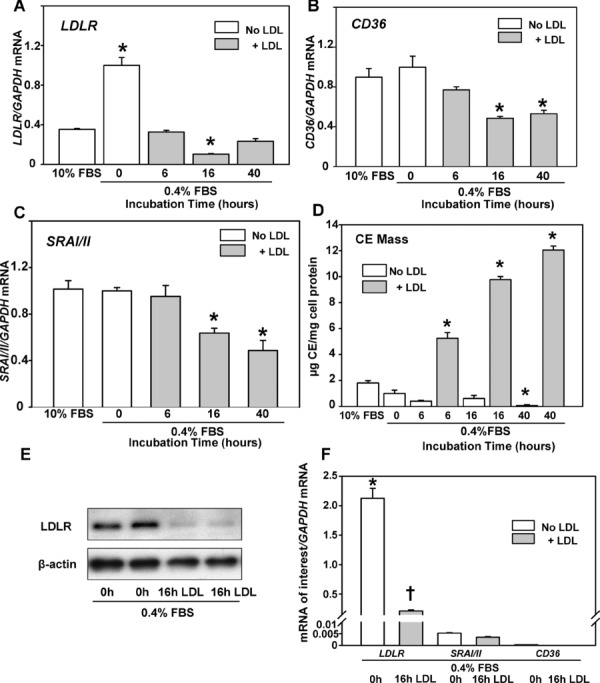
mRNA expression of *LDLR*, *CD36*, *SRAI/II*, CE content, and LDLR protein in human SMCs incubated with LDL. A through C, mRNA of SMCs incubated in M199 media containing 0.4% FBS (48 h) followed by incubation in M199 0.4% FBS with LDL (150 μg lipoprotein cholesterol / mL media) for up to 40 h. A, *LDLR*; B, *CD36*; and C, *SRAI/II*. Values for cells incubated in M199 0.4% FBS media without LDL are set at 1.0. Values for cells incubated in M199 10% FBS media are shown for comparison. Values are mean±SEM for 4 separate experiments. *P<0.05 vs cells grown in 10% FBS without LDL analyzed by the Mann-Whitney *U* nonparametric test. D, CE content of SMCs incubated in M199 0.4% FBS media (48 h) followed by incubation in M199 0.4% FBS media with or without LDL for up to 40 h. Values are mean±SEM for 4 separate experiments. *P<0.05 vs cells at 0 h, grown in 0.4% FBS without LDL as analyzed by the Student *t* test. E, Representative immunoblot of LDL receptor protein from postnuclear fractions of SMCs incubated as in A through C. F, Relative mRNA content of *LDLR*, *SRAI/II*, and *CD36* in SMCs incubated in M199 0.4% FBS media (48 h) followed by incubation in M199 0.4% FBS media with LDL (150 μg lipoprotein cholesterol/mL media) for 0 or 16 h. Values are mean±SEM for 3 separate experiments. *P<0.03 vs *LDLR* at 16 h and *SRAI/II* and *CD36* at 0 and 16 h of incubation with LDL; †*P*<0.03 vs *SRAI/II* and *CD36* at 0 and 16 h of incubation with LDL, as analyzed by the nonparametric Mann-Whitney *U* test.

The greater CE accumulation in cells exposed to LDL, compared to acLDL, oxLDL, or VLDL, was evaluated by determining the expression of cellular receptors. Under basal conditions (0.4% FBS, 48 h), *LDLR* expression was >40-fold higher than that of *SRAI/II*, *CD36,* and *SR-B1* (*P*<0.03) (Figure 4A). This difference was reflected in lipoprotein uptake. In SMCs incubated with DiI-LDL, DiI-fluoresence was increased 68-fold (*P*=0.01), whereas incubation with DiI-acLDL, DiI-fluoresence increased only 2-fold (*P*=0.001) (Figure 4B) and explains the enhanced ability of native LDL to induce SMC foam cell formation. Incubation of SMCs with LDL decreased *LDLR* mRNA at 6 h (−60%; *P*=0.029), at 16 h (−90%; *P*=0.029), and at 40 h (−75%; *P*=0.029) (Figure 5A), which was consistent with immunoblots showing low levels of LDLR protein, as assessed visually (Figure 5E). Following incubation with LDL, *SRAI/II* and *CD36* mRNA expression did not change after 6 h and decreased ≈30% after 16 h and 40 h (*P*<0.05 for both). However, their expression remained <2.5% that of *LDLR* (*P*<0.03 for both*)* (Figure 5B, 5C, and 5F). Although the *LDLR* became downregulated in response to LDL, significant CE accumulation had already occurred, as was observedat 6 h (5-fold; *P*=0.001), and continued to be elevated at 16 h (9-fold; *P*=0.001) and at 40 h (12-fold; *P*=0.001) (Figure 5D).

Incubation of cells with 24*(S)*,25-EC (1 μmol/L) decreased *LDLR* mRNA by 75% (*P*<0.001), LDLR protein by 60% (assessed visually), and DiI-LDL uptake by 90% (*P*<0.001) (Figure 4B, 4C, and 4E). In contrast, T0901317 (1 μmol/L) significantly increased *LDLR* mRNA 1.5-fold (*P*<0.001) yet decreased both LDLR protein (by 40%) and DiI-LDL uptake (by 50%) (*P*<0.001). The OSCi (20 nmol/L) significantly decreased *LDLR* mRNA by 50% (*P*<0.001), LDLR protein by 30%, and DiI-LDL uptake by 40% (*P*=0.017) (Figure 4B, 4C, and 4E). *LDLR* mRNA was unaffected by the OSCi at 100 nmol/L (Figure 4C).

**Figure 6.**
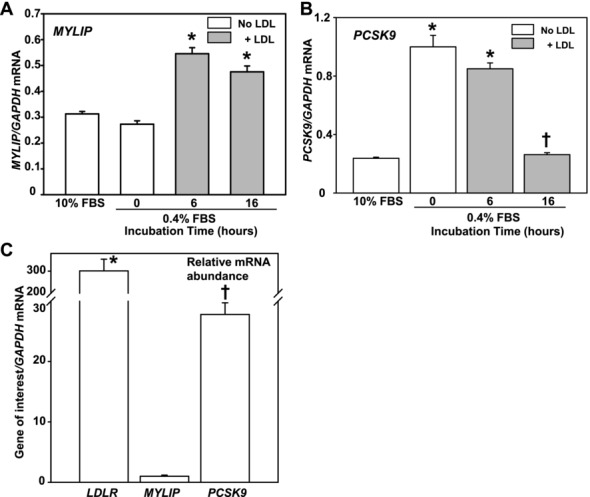
*MYLIP* and *PCSK9* mRNA expression in human SMCs incubated with LDL. mRNA content of SMCs incubated in M199 media containing 0.4% FBS (48 h) followed by incubation in M199 0.4% FBS media with or without LDL (150 μg lipoprotein cholesterol/mL media) for up to 16 h. A, *MYLIP* (n=3); B, *PCSK9* (n=3). Values are mean±SEM. *P<0.029 vs cells grown in 10% FBS media without LDL and †*P*=0.029 vs cells grown in 0.4% FBS media without LDL, as analyzed by the Mann-Whitney *U* nonparametric test. C, Relative mRNA content of *LDLR, MYLIP*, and *PCSK9* in SMCs incubated in M199 0.4% FBS media (48 h). Values are mean±SEM for 3 separate experiments. *P=0.002 vs *MYLIP* and *PCSK9* and †*P*=0.002 vs *MYLIP*, as analyzed by the Mann-Whitney *U* nonparametric test.

**Figure 7.**
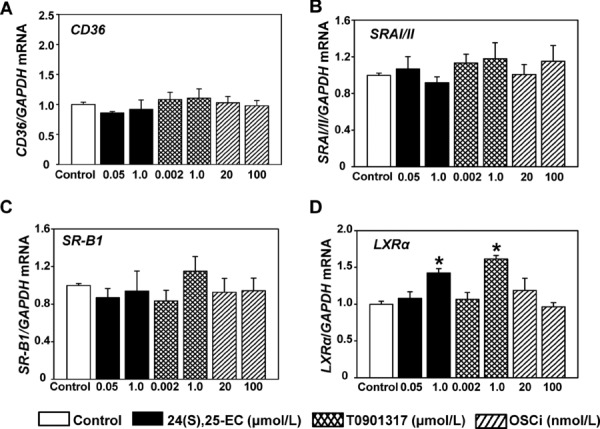
Effect of 24*(S)*,25-EC, T0901317, and OSCi on mRNA expression of *CD36*, *SRAI/II*, *SR-B1,* and *LXRα* in human SMCs. mRNA from SMCs incubated in M199 media containing 0.4% FBS (24 h) followed by incubation in M199 0.4% FBS media (basal) with vehicle alone, 24*(S)*,25-EC, T0901317, or OSCi for 24 h. Values for mRNA abundance of *CD36* (n=4) (A), *SRAI/II* (n=3) (B), *SR-B1* (n=5) (C), and *LXRα* (n=5) (D) were normalized to *GAPDH* mRNA abundance. Values are mean±SEM expressed as a ratio of vehicle alone. *P<0.05 vs control, as analyzed by the Mann-Whitney *U* nonparametric test.

Expression of *MYLIP* (or *IDOL*), a protein that induces ubiquitination of the LDLR and its lysosomal degradation, is regulated by LXR.^25^ Although *MYLIP* expression was low in SMCs (≈0.3% of the LDLR, *P*<0.002), as anticipated, its expression increased (≈2-fold; *P*=0.029) with LDL loading^25^ (Figure 6A and 6C). *MYLIP* mRNA was increased by 24*(S)*,25-EC (7-fold; *P*<0.001) and the OSCi at 20 nmol/L (3-fold; *P*=0.001) (Figure 4D), indicating that *MYLIP* likely contributed to the decrease in LDLR protein and LDL uptake (Figure 4B and 4E). T0901317 (1 μmol/L) increased *MYLIP* mRNA by 7-fold (*P*=0.008) (Figure 4D), which suggests a mechanism for the decreased LDL protein and LDL uptake by T0901317, despite increased *LDLR* mRNA. 24*(S)*,25-EC, T0901317, or the OSCi had no effect on the expression of *CD36*, *SRAI/II,* or *SR-B1* (Figure 7A through 7C) and did not change the modest increase in DiI-acLDL uptake (Figure 4B).

### 24*(S)*,25-EC and OSCi Reduce *SREBP-2, PCSK9,* and *HMGCR* Expression and Decrease Cholesterol Synthesis in SMCs

**Figure 8.**
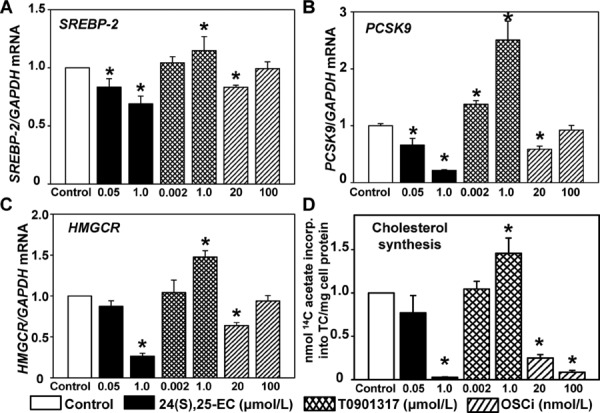
24*(S)*,25-EC and OSCi reduce cholesterol synthesis in human SMCs through reduction of SREBP-2–regulated gene expression. A through C, mRNA of *SREBP-2* (n=3) (A), *PCSK9* (n=4) (B), and *HMGCR* (n=4) (C), normalized to *GAPDH* in SMCs incubated in M199 media containing 0.4% FBS (24 h) followed by a 24-h incubation in M199 0.4% FBS media with or without vehicle, 24*(S)*,25-EC, T0901317, or OSCi. Values are mean±SEM relative to control. *P<0.05 vs control analyzed by the Mann-Whitney *U* nonparametric test. D, SMCs were incubated with compounds as above, followed by a further 5-h incubation with compounds plus [^14^C]-acetate to determine acetate incorporation into cholesterol. Values were normalized to control and are mean±SEM (n=3). *P<0.05 vs control analyzed by the Mann-Whitney *U* nonparametric test.

*SREBP-2* is regulated by oxysterols and controls the expression of *LDLR, PCSK9,* and *HMGCR*. 24*(S)*,25-EC (1 μmol/L) and the OSCi (20 nmol/L) decreased *SREBP-2* mRNA by 30% and 17%, respectively (*P*<0.001 for both) (Figure 8A), whereas T0901317 modestly increased *SREBP-2* mRNA (1.2-fold; *P*<0.05). The mRNA of *proprotein convertase subtilisin/kexin type 9 (PCSK9)* codes for a protein that targets the LDLR for lysosomal degradation.^26^ As anticipated, *PCSK9* mRNA was increased (5-fold; *P*=0.029) in SMCs incubated in 0.4% FBS media and was markedly reduced (−80%; *P*=0.029) with LDL-loading for 16 h (Figure 6B). 24*(S)*,25-EC and the OSCi at 20 nmol/L significantly decreased *PCSK9* mRNA by 80% and 40%, respectively (*P*<0.001 for both) (Figure 8B). Conversely, T0901317 (1 μmol/L) significantly increased *PCSK9* mRNA by 2.5-fold (*P*=0.002) (Figure 8B), revealing another mechanism for the reduced LDLR protein, decreased LDL uptake, and lower CE accumulation, despite increased *LDLR* mRNA (Figure 2B and Figure 4B, 4C, and 4E) in cells preincubated with T0901317. 24*(S)*,25-EC (1 μmol/L) decreased *HMGCR* mRNA by 70% (*P*<0.001) (Figure 8C) and cholesterol synthesis by 90% (*P*=0.036) (Figure 8D). The OSCi (20 nmol/L) significantly decreased *HMGCR* mRNA by 40% (*P*<0.001) and cholesterol synthesis by 70% (*P*=0.016). At 100 nmol/L, the OSCi decreased cholesterol synthesis by 90% (*P*=0.004) (Figure 8D) but did not affect *HMGCR* mRNA (*P*=NS) (Figure 8C) because of diminished 24*(S)*,25-EC (Figure 3). In contrast, T0901317 (1 μmol/L) significantly increased both *HMGCR* mRNA (1.5-fold; *P*<0.001) and cholesterol synthesis (1.4-fold; *P*=008) (Figure 8C and 8D).

### 24*(S)*,25-EC and T0901317 Increase the Expression of *LXRα, ABCA1,* and *ABCG1* and Enhance Cholesterol Efflux in SMCs

**Figure 9.**
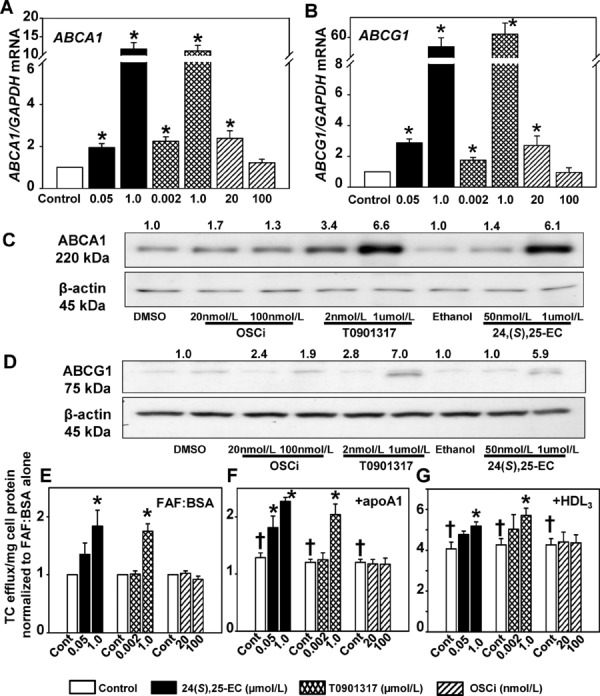
24*(S)*,25-EC, T0901317, and OSCi increase *ABCA1* and *ABCG1* and enhance cholesterol efflux in human SMCs. A and B, mRNA of *ABCA1* (A) and *ABCG1* (B), normalized to *GAPDH* from SMCs incubated in M199 media containing 0.4% FBS (24 h), followed by a 24-h incubation in M199 0.4% FBS media with or without vehicle, 24*(S)*,25-EC, T0901317, or OSCi. Values are mean±SEM relative to control for 4 separate experiments (n=4). *P<0.002 vs control as analyzed by the Mann-Whitney *U* nonparametric test. C and D, Representative immunoblots of ABCA1, ABCG1, and β-actin from postnuclear fractions of SMCs, incubated as in A and B. Mean values of protein band intensities, normalized to β-actin, are expressed relative to control for 5 separate experiments (n=5). E through G, SMCs were incubated in M199 media containing 0.4% FBS (24 h) followed by incubation (24 h) in M199 0.2% FAF:BSA media plus acLDL (5 μg TC) and [^3^H]cholesterol. Cells were then incubated with fresh FAF:BSA media with or without 24*(S)*,25-EC, T0901317, or OSCi for 24 h, followed by incubation with fresh FAF:BSA media with or without compounds and with or without apoA1 (10 μg/mL) or HDL_3_ (100 μg/mL) for 16 h. Values for cholesterol (TC) efflux (16 h) to FAF:BSA, apoA1, or HDL_3_ are mean±SEM and are relative to FAF:BSA alone for 5 separate experiments (n=5). *P<0.05 vs FAF:BSA (E), apoA1 (F), or HDL_3_ (G) alone, respectively. †*P*<0.05 vs FAF:BSA alone, as analyzed by Student *t* test.

T0901317 activates LXR in human coronary, aortic and bronchialSMCs.^11,13,27^ In human HITC6 SMCs, 24*(S)*,25-EC, OSCi (20 nmol/L), and T0901317 activated LXR as assessed by enhanced feed-forward *LXRα* expression (1.4- to 1.6-fold, *P*<0.05) (Figure 7D). Both 24*(S)*,25-EC (1 μmol/L) and T0901317 (1 μmol/L) increased *ABCA1* mRNA 12-fold (*P*<0.001 for both) and increased *ABCG1* expression, which was very low under basal conditions, ≈60-fold (*P*=0.002 for both) (Figure 9A and 9B). The OSCi (20 nmol/L) increased *ABCA1* and *ABCG1* mRNA 2.5- and 3.0-fold (*P*<0.001 for both), respectively, whereas OSCi (100 nmol/L) had no effect (*P*=NS) (Figure 9A and 9B). Immunoblot analysis demonstrated that 24*(S)*,25-EC (1 μmol/L) and T0901317 (1 μmol/L) increased ABCA1 and ABCG1 protein by ≈6- to 7-fold, as assessed visually (Figure 9C and 9D). The OSCi (20 nmol/L) increased ABCA1 and ABCG1 levels 1.7- and 2.4-fold, respectively. 24*(S)*,25-EC (1 μmol/L) and T0901317 (1 μmol/L) stimulated cholesterol efflux to FA-free BSA (FAF:BSA) (*P*<0.01 for both) (Figure 9E). Addition of apoAI significantly increased efflux by 30% (*P*<0.001) compared to FAF:BSA alone, which was stimulated further (≈2-fold) by 24*(S)*,25-EC (1 μmol/L) or T0901317 (1 μmol/L) (*P*<0.01 for both) (Figure 9F). Cholesterol efflux to HDL_3_ was increased 4-fold (*P*=0.004) compared to FAF:BSA alone (Figure 9G). 24*(S)*,25-EC or T0901317 further increased efflux to HDL_3_ (1.4-fold; *P*<0.05 and *P*<0.01, respectively) compared to HDL_3_ alone. The OSCi did not affect cholesterol efflux, which suggests that the modest increases in *ABCA1*and *ABCG1* expression were insufficient to stimulate efflux.

### T0901317 but Not 24*(S)*, 25-EC or OSCi Stimulates *SREBP-1c* Expression, SREBP-1 Processing, and Lipogenesis in SMCs

**Figure 10.**
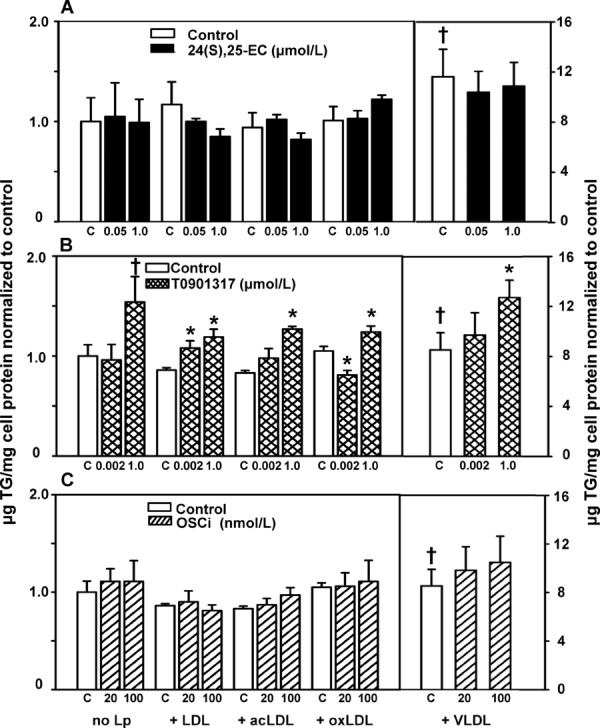
24*(S)*,25-EC and OSCi do not increase triglyceride mass in human SMCs. A through C, After 24-h preincubation in M199 media containing 0.4% FBS, SMCs were incubated in the same media with or without vehicle, 24*(S)*,25-EC (A), T0901317 (B), or OSCi (C) for 24 h followed by incubation for 16 h in M199 0.4% FBS media with or without compounds and with or without LDL, acLDL, oxLDL (150 μg cholesterol/mL), or VLDL (50 μg cholesterol/mL). Values for triglyceride (TG), normalized to no lipoprotein (Lp), are mean±SEM for 4 separate experiments (n=4). *P<0.05 vs control plus lipoprotein and †*P*<0.05 vs control with no Lp, as analyzed by Student *t* test.

SMCs incubated with T0901317 and adipocyte differentiation media stimulate SREBP-1c–mediated FA synthesis, resulting in a foam cell phenotype.^13^ In human HITC6 SMCs, incubation with LDL, acLDL, or oxLDL did not affect triglyceride mass, but incubation of cells with VLDL increased triglyceride mass >8.0-fold (*P*=0.01) (Figure 10A through 10C and Figure 1). 24*(S)*,25-EC or the OSCi did not affect triglyceride in SMCs incubated with or without lipoproteins (Figure 10A and 10C). In contrast, T0901317 increased triglyceride mass up to 1.5-fold in the absence or presence of LDL, acLDL, or oxLDL (*P*<0.05 for each) (Figure 10B) and increased triglyceride mass to an even greater extent (60%; *P*=0.023) than the 8-fold induction by VLDL alone (Figure 10B).

**Figure 11.**
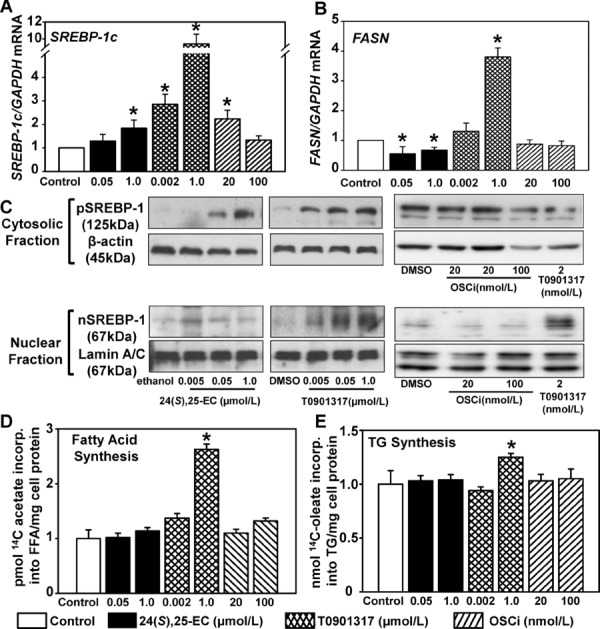
24*(S)*,25-EC, T0901317, and OSCi increase *SREBP-1c mRNA* in human SMCs, but only T0901317 increases FA and triglyceride synthesis. A and B, After 24-h preincubation in M199 media containing 0.4% FBS, SMCs were incubated (24 h) in M199 0.4% FBS media with or without vehicle, 24*(S)*,25-EC, T0901317, or OSCi. Values for mRNA of *SREBP-1c* (n=4) (A) and *FASN* (n=3) (B), normalized to *GAPDH,* are mean±SEM relative to control. *P<0.001 vs control, as analyzed by the Mann-Whitney *U* nonparametric test. C, SMCs were treated as in A and B. Representative immunoblots are of precursor (p) and nuclear (n) SREBP-1 from SMC postnuclear and nuclear fractions, respectively (n=3). The nSREPB-1/Lamin A/C blots in C (right) were derived from gels run for a longer time (≈180 min) compared to the other nSREPB-1 / Lamin A/C blots (≈120 min) (left and center). D, [^14^C]acetate incorporation into FA (5 h) in SMCs preincubated as in A and B. Values were normalized to control for 4 separate experiments (n=4) and are mean±SEM. *P<0.008 vs control as analyzed by the Mann-Whitney *U* nonparametric test. E, [^14^C]oleate incorporation into triglyceride (5 h) in SMCs incubated as in D. Values are normalized to control and are mean±SEM for 5 separate experiments (n=5). *P<0.05 vs control, as analyzed by Student *t* test.

We determined whether 24(*S*),25-EC blocks SREBP-1 processing, thereby preventing lipogenesis and triglyceride accumulation. In HITC6 SMCs, 24*(S)*,25-EC (1 μmol/L) and OSCi (20 nmol/L) stimulated moderate increases in *SREBP-1c* mRNA (2-fold; *P*=0.001 for both), whereas the OSCi at 100 nmol/L had no effect (*P*=NS) (Figure 11A). In contrast, T0901317 (1 μmol/L) markedly increased *SREBP-1c* mRNA by 10-fold (*P*<0.001). *FASN* mRNA was modestly decreased by 24*(S),*25-EC (−40%; *P*<0.001), and the OSCi had no effect(*P*=NS) (Figure 11B), but T0901317 (1 μmol/L) increased *FASN* expression 4-fold (*P*<0.001).

Cleavage of pSREBP-1c to nSREBP-1c is required for activation of target genes. Visual inspection of immunoblots revealed that 24*(S)*,25-EC and OSCi (20 nmol/L) modestly increased pSREBP-1 levels, whereas nSREBP-1 levels were unaffected (Figure 11C). In contrast, T0901317 enhanced pSREBP-1 and markedly increased levels of nSREBP-1. To assess the functional significance of increased *SREBP-1c* mRNA, nSREBP-1 protein, and *FASN* mRNA, the synthesis of FA and triglyceride was determined. Neither 24*(S)*,25-EC nor OSCi altered FA or triglyceride synthesis (*P*=NS) (Figure 11D and 11E). In contrast, T0901317 (1 μmol/L) significantly increased FA synthesis by 2.8-fold (*P*=0.008) and triglyceride synthesis by 1.3-fold (*P*=0.05) (Figure 11D and 11E).

### 24*(S)*,25-EC Restores SMC Assembly of Fibonectin and Collagen Fibrils

**Figure 12.**
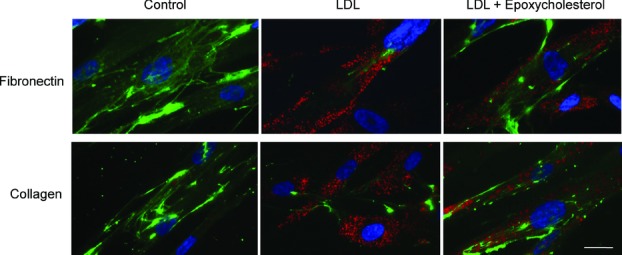
24*(S*),25-EC preserves SMC-mediated fibronectin and collagen fibril assembly in the presence of LDL. Fluorescent micrographs of HITC6 SMCs initially incubated in M199 media containing 0.4% FBS for 24 h and subsequently incubated (24 h) in M199 0.4% FBS media with or without vehicle or 1 μmol/L 24*(S*),25-EC. Cells were then incubated for a further 24 h in fresh media with or without 1 μmol/L 24*(S*),25-EC and with or without LDL (150 μg cholesterol/mL) plus 100 nmol/L Oregon Green–labeled human fibronectin protomers (top row) or 1 μg/mL Oregon Green–labeled solubilized rat tail collagen (bottom row). Fixed cells were stained with Oil Red O to identify cytoplasmic lipid droplets. Green signals depict fibronectin or collagen that has been assembled into fibrils by the SMCs. Nuclei were stained with 2.5 μg/mL Hoechst 33258. Bar=20 μm.

We assessed whether SMC-dependent functions were retained by 24(*S*),25-EC in SMCs exposed to LDL. Elaboration of extracellular matrix was evaluated by examining microscopically the assembly of fibronectin and type I collagen fibrils in SMCs after the addition of labeled soluble precursors of either fibronectin or collagen. SMCs not exposed to LDL assembled an elaborate network of both fibronectin and collagen fibrils (Figure 12). Incubation of SMCs with LDL resulted in a marked decrease in their ability to assemble these fibrils, commensurate with increased Oil Red O–stained lipid, as we reported previously.^5^ In contrast, preincubation of cells with 24(*S*),25-EC, followed by the addition of LDL, decreased Oil Red O–stained lipid and preserved the ability of SMCs to assemble both fibronectin and collagen.

## Discussion

Human SMC–derived foam cells promote atherosclerosis; they are observed within the musculoelastic layer at early stages and within the intimal fibrotic layer of intermediate and advanced lesions.^1^ Our results revealed that in human SMCs, native LDL was the predominant lipoprotein responsible for the induction of CE accumulation, whereas uptake of modified LDL was limited. This differencecorrelated with greater *LDLR* expression than scavenger receptor expression. Furthermore, we show for the first time that 24*(S)*,25-EC attenuates CE accumulation in SMCs challenged with human LDL. Reduced LDL uptake by 24*(S)*,25-EC was linked to inhibition of SREBP-2–mediated *LDLR* expression. 24*(S)*,25-EC also increased *MYLIP* expression, which contributed to the decrease in LDLR protein and LDL uptake. 24*(S)*,25-EC stimulated cholesterol efflux via activation of *ABCA1* and *ABCG1*, further reducing intracellular cholesterol. The impact of endogenously synthesized 24*(S)*,25-EC was qualitatively similar. Importantly, with exposure of SMCs to 24*(S)*,25-EC, in contrast to the nonsteroidal LXR agonist T0901317, cholesterol efflux was selectively increased without inducing a triglyceride-rich foam cell phenotype.

Recently, we demonstrated that human SMCs display impaired assembly of type I collagen and fibronectin after lipid accumulation induced by LDL or VLDL.^5^ These findings directly link SMC foam cell formation with failure to elaborate extracellular matrix, potentially accounting for reduced plaque stability. These human SMCs, the same clone as those used in the present study, maintained expression of SMC-specific markers upon lipid loading.^5^ We now show that attenuated LDL-induced CE accumulation and enhanced cholesterol efflux by 24*(S)*,25-EC preserves the assembly of fibrillar collagen and fibronectin, a characteristic of a more productive SMC phenotype.

CE accumulation in SMCs challenged with acLDL or oxLDL was substantially lower than in those challenged with LDL (1.5- versus 6-fold; *P*<0.05). These human SMCs expressed more *LDLR* (>40-fold higher) than *CD36* and *SRAI/II,* consistent with previous reports that scavenger receptor expression was undetectable in intimal or medial SMCs within human atherosclerotic lesions.^28^ In cultured rabbit aortic SMCs, uptake of modified lipoproteins was low, whereas CE accumulation was derived from native LDL.^4^ Our results provide direct evidence that CE accumulation in human SMCs is primarily a consequence of LDL uptake. This differs from macrophages, in which modified lipoproteins primarily drive foam cell formation.^29^ Furthermore, our results extend the concept that VLDL-induced triglyceride accumulation in SMCs results in a foam cell–like phenotype.^5,7^

Expression of *LDLR, PCSK9,* and *HMGCR* is primarily regulated by the nuclear form of SREBP-2.^18,30^ We demonstrated that SMCs exposed to exogenous or endogenous 24*(S)*,25-EC contained less *SREBP-2* and *LDLR* mRNA, consistent with diminished *LDLR* expression in macrophages^15^ and fibroblasts^31^ incubated with 24*(S)*,25-EC. The mechanism involved oxysterol-mediated disruption of pSREBP-2 maturation, preventing *LDLR* expression and the feed-forward amplification of *SREBP-2* transcription.^19,31,32^ The present study is the first to report that 24*(S)*,25-EC in human SMCs decreased expression of SREBP-2–responsive genes, *SREBP-2* and *LDLR*, and increased the expression of *MYLIP,* resulting in blunted *LDLR* expression and the attenuation of LDL uptake and CE accumulation.

The SREBP-2–mediated expression of *PCSK9*^32^ was also decreased by 24*(S)*,25-EC in SMCs. As PCSK9 targets the LDLR for lysosomal degradation,^26,30^ decreased *PCSK9* expression would be expected to impede LDLR degradation.^26,30^ The ability of 24*(S)*,25-EC to also decrease SREBP-2–mediated *LDLR* mRNA when combined with the increased LXR-stimulated *MYLIP* expression, which would promote LDLR degradation,^25^ resulted in attenuation of LDLR protein and LDL uptake. 24*(S)*,25-EC also inhibited *HMGCR* expression, providing further evidence that 24*(S)*,25-EC prevented pSREBP-2 maturation.^31^ As oxysterols are known to increase HMGCR enzyme degradation,^33^ the reduced transcription and increased degradation of HMGCR provided a plausible mechanism for the marked reduction of cholesterol synthesis in 24*(S)*,25-EC–treated SMCs.

Partial inhibition of OSC reduced cholesterol synthesis while maximizing 24*(S)*,25-EC synthesis.^15,16^ In SMCs, endogenous 24*(S)*,25-EC inhibited the expression of *SREBP-2, LDLR,* and *PCSK9* and increased *MYLIP* expression, collectively resulting in diminished LDLR-mediated LDL uptake. Although the effect of OSCi was less marked, likely because maximal synthesis of 24*(S)*-25-EC was lower than optimal concentrations of exogenous 24*(S)*,25-EC, reduced LDL uptake remained significant (*P*=0.017).

In contrast to 24*(S)*,25-EC, T0901317 increased *SREBP-2, LDLR, PCSK9,* and *HMGCR* expression, which led to enhanced cholesterol synthesis yet a significant decrease in LDL uptake (*P*<0.001). T0901317, a nonsteroid, did not inhibit pSREBP-2 maturation and thus stimulated expression of SREBP-2–responsive genes. Enhanced *LDLR* and *PCSK9* expression have been observed in T0901317-treated hepatocytes.^25,34^ Although the *LDLR* promoter contains an LXR response element,^35^ expression is significantly more responsive to sterol response element activation, which provides an explanation for increased *LDLR* expression by T0901317 but not by 24*(S)*,25-EC. In the present study, T0901317 markedly induced *SREBP-1c* expression and nSREBP-1 protein, similar to a previous report in SMCs.^13^ In contrast to 24*(S)*,25-EC, the T0901317-induced overabundance of nSREBP-1 likely increased nonselective binding to SREs and activation of both *SREBP-1c–* and *SREBP-2–*target genes. This has been demonstrated in T0901317-treated Huh7 cells^35^ and in tissues in which nSREBPs are overexpressed.^36^

*PCSK9* expression is primarily regulated by SREBP-2; however, a recent report revealed marked SREBP-1c stimulation of *PCSK9* mRNA in T0901317-treated hepatoctyes.^32,34^ Although an LXRresponse element has not been identified in the *PCSK9* promoter, the increase we observed in *PCSK9* mRNA in T0901317-treated SMCs but not in 24*(S)*,25-EC–treated SMCs indicated that *PCSK9* expression was highly responsive to nSREBP-1c. Despite increased *LDLR* mRNA by T0901317, the ability of T0901317 to also increase the expression of both *MYLIP*^25^ and *PCSK9* resulted in diminished LDLR protein and decreased LDL uptake.

Activation of ABCA1 and ABCG1 and cholesterol efflux by synthetic LXR agonists has been documented in rat aortic SMCs^27^ and human coronary^12^ and airway^11^ SMCs. Furthermore, T0901317-induced cholesterol efflux in human airway SMCs was mediated exclusively by ABCA1.^11^ In the present study, we demonstrate in HITC6 SMCs that exogenous 24*(S)*,25-EC increased *LXRα, ABCA1,* and *ABCG1* expression, which led to enhanced cholesterol efflux. These observations are consistent with our previous studies in macrophages.^15,16^ Furthermore, we show that 24*(S)*,25-EC–mediated cholesterol efflux contributed to the attenuation of SMC foam cells. Endogenous synthesis of 24*(S)*,25-EC, induced by partial OSC inhibition, increased *ABCA1* and *ABCG1* expression to levels ≈20% of those in cells exposed to exogenous 24*(S)*,25-EC yet did not increase cholesterol efflux. Therefore, although SMCs synthesize endogenous 24*(S)*,25-EC, LXR-induced gene expression was likely below the threshold for facilitating cholesterol removal.

LXR has been shown to regulate de novo FA biosynthesis through expression of *SREBP-1c* and *FASN*.^14^ T0901317 stimulated FA synthesis and triglyceride accumulation in cells and mice.^9,15,16^ In human SMCs, T0901317 increased de novo FA synthesis when incubated in adipocyte differentiation media.^13^ Increased lipogenesis appears to be exclusive to nonsteroidal LXR agonists such as T0901317, because oxysterols such as 24*(S)*,25-EC and 22*(R)*-OH did not affect lipogenesis in macrophages.^15,16^ Our results clearly show that in human SMCs, 24*(S)*,25-EC did not stimulate *SREBP-1c* or *FASN* mRNA and did not increase nSREBP-1 protein or synthesis of FA and triglyceride. Furthermore, triglyceride accumulation in SMCs exposed to lipoproteins was unaffected, whereas T0901317 stimulated FA synthesis and amplified triglyceride accumulation, especially in SMCs incubated with VLDL. This is consistent with a mechanism in which 24*(S)*,25-EC, but not T0901317, retains pSREBP-1c within the endoplasmic reticulum, preventing nSREBP-1 formation and nSREBP-1c–stimulated gene expression, including the feed-forward activation of *SREBP-1c* itself.^37^ Thus, 24*(S)*,25-EC acts as a safety valve to diminish cellular cholesterol and prevent excessive FA synthesis and triglyceride storage. In recent studies, synthetic oxysterol-like LXR agonists reduced atherosclerosis in mice but, like steroidal agonists, did not induce FA synthesis.^38^ Therefore, the present study in SMCs and our previous studies in macrophages^15,16^ demonstrate that 24*(S)*,25-EC has the potential to selectively activate LXR-regulated cholesterol efflux from cells involved in atherogenesis, without inducing lipogenesis.

SMCs participate in plaque development and have the propensity to develop into foam cells through acquisition of lipoprotein-derived lipids. Controlling cholesterol homeostasis in SMCs represents an attractive mechanism to maintain SMCs in a reparative phenotype. Exposure of human SMCs to the oxysterol LXR agonist 24*(S)*,25-EC reduced native LDL uptake, the primary mechanism for SMC foam cell formation. Furthermore, 24*(S)*,25-EC promoted cholesterol efflux without inducing lipogenesis. Diversion of SMCs from a foam cell state restored their ability to elaborate extracellular matrix, which has the potential to enhance lesion stabilization.
